# Situational Awareness: Regulation of the Myb Transcription Factor in Differentiation, the Cell Cycle and Oncogenesis

**DOI:** 10.3390/cancers6042049

**Published:** 2014-10-02

**Authors:** Olivia L. George, Scott A. Ness

**Affiliations:** Department of Internal Medicine, Section of Molecular Medicine, University of New Mexico Health Sciences Center, MSC07 4025—CRF 121, 1 University of New Mexico, Albuquerque, NM 87131, USA; E-Mail: ogeorge@hawaii.edu

**Keywords:** chromatin, gene regulation, cyclins, post-translational modifications, cancer, salivary gland, epithelia, differentiation, RNA splicing, signaling

## Abstract

This review summarizes the mechanisms that control the activity of the c-Myb transcription factor in normal cells and tumors, and discusses how c-Myb plays a role in the regulation of the cell cycle. Oncogenic versions of c-Myb contribute to the development of leukemias and solid tumors such as adenoid cystic carcinoma, breast cancer and colon cancer. The activity and specificity of the c-Myb protein seems to be controlled through changes in protein-protein interactions, so understanding how it is regulated could lead to the development of novel therapeutic strategies.

## 1. Introduction

Myb proteins are a family of transcription factors with highly conserved DNA binding domains that are found in insects, higher plants and vertebrates, that are often involved in the regulation of differentiation, proliferation or both, and that are implicated in many types of tumors, as discussed in several detailed reviews [1,2,3,4,5,6,7,8]. Differentiation and proliferation are often considered to be opposite outcomes: tumor cells fail to differentiate completely (or de-differentiate as in the case of epithelial to mesenchymal transition) and continue to proliferate or express stem cell-like features that keep them immortal and proliferating. Since Myb proteins are implicated in the regulation of both differentiation and proliferation, they may play important roles in deciding whether cells progress through the cell cycle and proliferate or instead arrest and differentiate. Since some successful chemotherapeutic strategies involve triggering the terminal differentiation of tumor cells that have become blocked in differentiation [9,10], Myb proteins would seem to be excellent targets for the development of novel therapeutic strategies that could shift tumor cells out of proliferation and into differentiation. However, the dual and conflicting roles of Myb proteins in processes that are often considered opposite raises questions about how the activities of Myb proteins are regulated and what types of interventions could be used to switch their activities in tumor cells from bad (inducing proliferation) to good (inducing differentiation). This review will focus on the functions of Myb proteins in regulating the proliferation and differentiation and how their activities could be regulated to induce tumor cells to differentiate.

### 1.1. Structures and Functions of Myb Proteins

**Figure 1:**
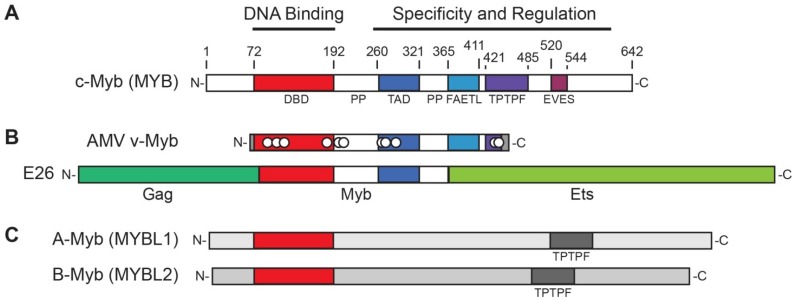
Myb Protein Structures and Conserved Domains. (**A**) Conserved domains in c-Myb. The structure of the c-Myb protein is diagrammed, with N-terminus at left and C-terminus at right. Domains that are most conserved in human, mouse and chicken proteins are shaded and amino acid residues are numbered above the diagram, including the DNA binding domain (red) near the N-terminus. Labels below the diagram indicate conserved domains (DBD, TAD, FAETL, TPTPF and EVES) and proline-rich regions (PP) that are discussed in the text; (**B**) Structures of the oncogenic v-Myb proteins encoded by Avian Myeloblastosis Virus (AMV) and E26 virus. The AMV protein has 6 amino acids derived from the retroviral Gag protein fused to amino acids 72-442 of c-Myb, fused to 13 novel non-Myb amino acids at the C-terminus (shaded gray). It also has eleven point mutations that distinguish it from c-Myb, indicated by white dots. The E26 protein is a Gag-Myb-Ets fusion, as indicated (the non-Myb regions are shaded dark and light green, respectively); (**C**) Diagrams of the structures of the related A-Myb (MYBL1) and B-Myb (MYBL2) proteins. These proteins are most similar to c-Myb in the conserved DNA binding domains (red) and in the conserved TPTPF domains (gray, labeled).

The MYB proto-oncogene encodes a transcription factor (c-Myb) with a conserved N-terminal DNA binding domain (Figure 1A) and several highly conserved domains [7] that are involved in transcriptional activation, specificity and negative regulation. The MYB gene is the normal cellular counterpart to the *v-*Myb (viral-Myb) oncogenes found in two chicken leukemia viruses, Avian Myeloblastosis Virus (AMV) and E26 virus (Figure 1B). Both AMV and E26 transform immature hematopoietic cells in tissue culture and induce myeloid leukemias in chickens [1]. The v-Myb and c-Myb (cellular Myb) proteins share a highly conserved DNA binding domain near the N-terminus, which is also found in the related proteins A-Myb (MYBL1) and B-Myb (MYBL2) (Figure 1C). A second conserved domain, labeled “TPTPF” in Figure 1, is also shared by the c-Myb, A-Myb and B-Myb proteins, but has an unknown function. All the Myb proteins are DNA-binding transcription factors that can recognize similar DNA sequences *in vitro* and that can activate the same reporter gene constructs in transfection assays [11,12]. Compared to c-Myb, the AMV v-Myb protein has truncations at both the N- and C-terminal regions and has eleven point mutations that cause amino acid changes. Several types of biological and gene activation assays have shown that all of the mutations acquired by v-Myb contribute to its oncogenicity and its distinct transcriptional activity, compared to c-Myb [13,14,15]. The E26 virus expresses a more complex version of v-Myb with 272 amino acids of the retroviral Gag protein fused to Myb, which is fused in turn to 491 amino acids from another transcription factor, Ets-1, at the C-terminus (Figure 1B) [1,7]. In each case, the C-terminal truncations are important for oncogenic activity, suggesting that the C-terminal domains of c-Myb suppress transforming activity [16,17].

The C-terminal region of c-Myb contains several domains that are highly conserved in the chicken, mouse and human proteins (Figure 1). These include the minimal transcriptional activation domain (TAD) required for activation of gene expression [18,19], the “FAETL” domain that is required for oncogenic activity [20], the “TPTPF” domain conserved in the other Myb proteins, and the “EVES” domain that is involved in intra-molecular interactions and negative regulation [21]. There are also proline-rich regions that may be involved in conformational changes catalyzed by peptidyl-prolyl isomerases [22,23]. Thus, the large C-terminal domain has multiple functional components involved in regulating both the specificity and the activity of c-Myb.

#### 1.1.1. Effects of DNA Binding Domain Mutations in v-Myb

The first evidence that Myb protein activity is regulated through protein-protein interactions came from studying the first known Myb-regulated gene, *mim-1*, which was identified by screening for genes that were activated by a temperature-sensitive version of the E26 v-Myb protein [24]. The *mim-1* gene was activated by the normal c-Myb and by the v-Myb protein from E26 virus, but not by the v-Myb protein encoded by Avian Myeloblastosis Virus, AMV (Figure 1). All three proteins were able to bind the *mim-1* promoter and to activate plasmid-born reporter gene constructs containing the *mim-1* gene promoter, but AMV could not activate the endogenous *mim-1* gene embedded in cellular chromatin [25]. The key differences between the AMV and c-Myb/E26 proteins were mapped to three point mutations in the DNA binding domain of the AMV-encoded protein [25].

When the solution structures [26,27] and then the crystal structures [28] of the Myb DNA binding domain bound to DNA became available, it became clear that the three amino acid changes (Figure 1) that made AMV v-Myb unable to activate the chromatin-embedded *mim-1* gene were on the outside surface of the DNA binding domain, facing away from the DNA. The amino acid changes were unable to directly affect the interaction of the protein with the DNA, and were instead likely to affect protein-protein interactions [7]. Thus, individual mutations in the DNA binding domain probably affect protein-protein interactions, rather than protein-DNA interactions, and lead to differences in which genes can be activated.

The Myb DNA binding domain has recently been grouped with conserved domains from several chromatin-remodeling enzymes that are collectively dubbed SANT domain proteins [29]. All of the MYB/SANT domain proteins are thought to bind histone tails and may play important roles in histone remodeling [30,31,32]. The other SANT domain proteins have enzymatic activities that play roles in chromatin remodeling. However, Myb is not known to have any enzymatic activities so its role in chromatin remodeling is likely to be as a regulator or “pioneer” transcription factor rather than as a catalyst of chromatin structure change [33,34]. Interestingly, the normal and oncogenic versions of the Myb DNA binding domain, which differ by the same surface residue mutations that affect activation of the *mim-1* gene, interact differently with histones, which could be a hint about how mutations unmask the oncogenic activity of Myb [35]. So the DNA binding domain mutations in v-Myb may affect its ability to alter chromatin structure, either directly or via protein-protein interactions with other chromatin remodeling enzymes, which could in turn affect which genes it regulates.

#### 1.1.2. Microarray Assays Uncover the Complexity of Myb Activities

The results with the *mim-1* gene pointed out the importance of protein-protein interactions in the regulation of Myb activity. But the magnitude of the changes that can occur in Myb protein activities became apparent when microarray experiments were used to compare the activities of c-Myb and v-Myb. Adenoviruses were used to express normal c-Myb or oncogenic v-Myb in human cells then microarrays were used to measure changes in gene expression. The expected result was that the two proteins would have similar activities, although v-Myb was expected to be more active since it lacks the C-terminal domain involved in negative regulation [22,36,37,38]. Instead, when c-Myb and v-Myb were expressed in normal human monocytes, the microarray assays showed that the two proteins activated different sets of target genes, as if they were two unrelated transcription factors [39]. Subsequent domain swap experiments showed that, although the differences in the DNA binding domains affected a few genes, the differences outside the DNA binding domain, in protein-protein interaction domains, were largely responsible for the different transcriptional activities of c-Myb and v-Myb [11,15]. Thus, although some genes like *mim-1* are affected by DNA binding domain mutations, Myb specificity was predominantly controlled by protein-protein interactions that occur outside the DNA binding domains [1,5]. A similar conclusion came from comparing the activities of A-Myb, B-Myb and c-Myb in microarray assays. Each type of Myb protein activated a different set of human genes, and domain swap experiments showed that the DNA binding domains were interchangeable, while the unique C-terminal parts of the proteins determined which target genes were affected [11,12].

Although all the Myb proteins share a common structure and have highly related DNA binding domains, they have different activities. The three normal proteins, c-Myb, A-Myb and B-Myb, have different tissue distributions and knock-out mutations lead to different outcomes, suggesting that each plays a unique biological role [1]. In addition, as discussed above, they each activated different sets of genes when ectopically expressed in human cells. Finally, only the v-Myb derivatives are oncogenic—the full-length, normal c-Myb protein fails to transform hematopoietic cells in tissue culture or to induce tumors or leukemias in animals [17,38]. These types of results led to an oft-cited hypothesis that the oncogenic v-Myb proteins represent a constitutively activated version of c-Myb [7]. This model fit with findings linking the C-terminal domains of c-Myb to decreased protein stability, and to auto-regulatory interactions that appeared to work through intramolecular interactions involving the N- and C-terminal domains of c-Myb, both of which are negated in v-Myb, since it has a C-terminal truncation [21]. However, several types of results have demonstrated that each Myb protein has unique activities and regulates distinctive sets of target genes, suggesting that differences in protein-protein interactions steer the Myb proteins to specific target genes [5].

**Figure 2:**
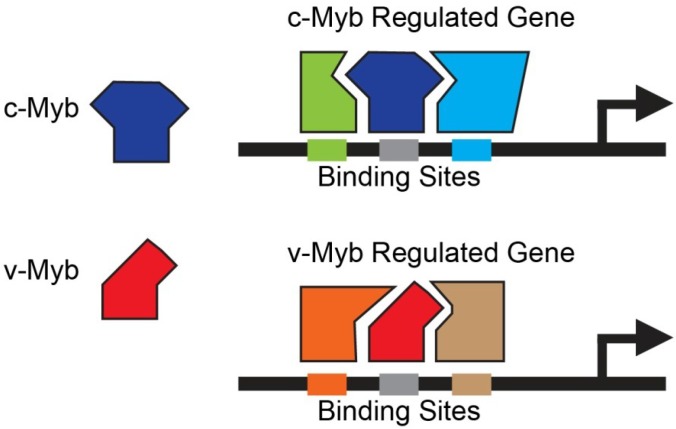
Myb Protein Interactions. This “transcription factor code” model depicts how c-Myb and v-Myb could be directed to different sets of target genes, if they interact with different co-regulators or co-activators. The DNA binding domains are interchangeable, but differences in protein-protein interactions stabilize the two Myb proteins on different sets of promoters.

To explain the differences in activities of different Myb proteins that share very similar DNA binding domains we proposed a transcription factor code model, in which protein-protein interactions at promoters play an important role in determining the specificities of the Myb proteins [5]. While the DNA binding domains are required to make contacts with specific sequences in the chromatin, the protein-protein interactions are important for stabilizing the binding of Myb proteins at the promoters of regulated genes. Different protein-protein interactions mediated by changes or mutations in the C-terminal domains of the Myb proteins or through post-translational modifications, would lead to the formation of stable Myb protein complexes at different promoters, and to different genes being regulated. The transcription factor code model (Figure 2) proposes that c-Myb and v-Myb interact with different co-regulators, which leads to them being stabilized at different promoters so they can activate different genes. Thus, changes in Myb that affect which proteins or co-regulators it interacts with could determine whether it has a normal or oncogenic activity and whether it induces proliferation or differentiation.

### 1.2. Mechanisms of Myb Activation in Cancer

The oncogenic v-Myb proteins were originally discovered in avian acute leukemia viruses. But mutations in the normal MYB gene have also been found in several types of human cancer. The MYB gene is frequently duplicated or amplified in a subset of pediatric T-cell acute lymphocyte leukemias (T-ALL) [1,40,41], suggesting that Myb protein over-expression contributes to transformation. There is also growing evidence that Myb proteins play important oncogenic roles in a variety of solid tumors. High expression of MYB transcripts has been found in pancreatic tumors, colon tumors, and estrogen receptor-positive breast cancers [1,4], and studies using MCF7 breast cancer cell lines showed that the MYB gene is directly regulated by estrogen receptor, linking c-Myb protein activity to estrogen-dependent proliferation [42]. However, depletion of c-Myb expression in MCF7 cells can lead to increased tumorigenesis *in vitro* and *in vivo*, suggesting that c-Myb may also have some tumor suppressing activity [43]. This is consistent with the long recognized dual role of Myb proteins in the regulation of both oncogenic proliferation and anti-oncogenic differentiation [25,44,45]. Thus, when Myb promotes proliferation it can be oncogenic, but when it promotes differentiation it can be anti-oncogenic. The question is: what turns Myb into an oncoprotein?

**Figure 3:**
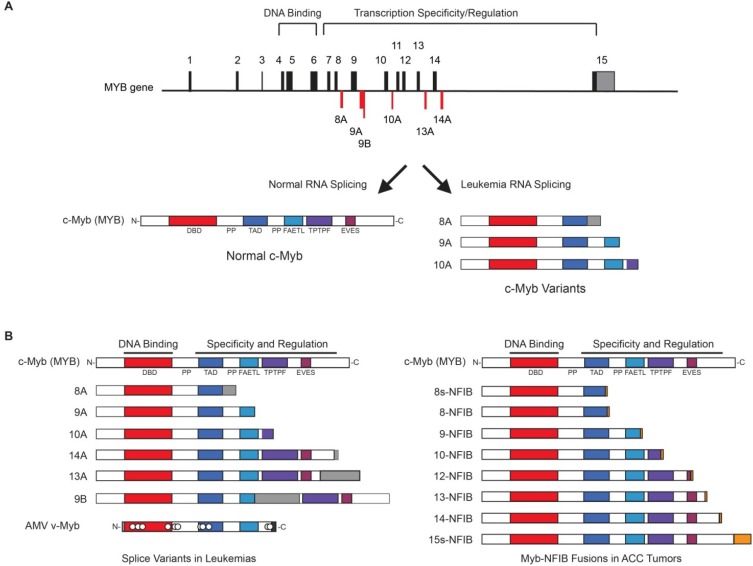
Different Tumor Mechanisms Generate Similar Myb Proteins. (**A**) Myb alternative RNA splicing. The exon/intron structure of the human MYB gene is shown at top, with 15 normal exons shown above the line and 6 alternatively spliced exons (red) shown below the line. Normal splicing generates mRNA that encodes the c-Myb protein (below left), but alternative splicing in leukemias leads to the expression of variant forms (below right), some of which have been linked to poor outcomes [46]; (**B**) Myb variants expressed in leukemias and solid tumors. Enhanced levels of alternative RNA splicing in leukemias generates variant forms of c-Myb protein (left) that correlate with poor outcome and could be oncogenic. In adenoid cystic carcinoma (ACC) with translocation t(6;9), the MYB gene is fused to the NFIB gene, generating fusion proteins (right) that resemble the variants from leukemias. Different mechanisms in leukemias and ACC tumors lead to the expression of similar truncated forms of c-Myb. Please see the legend to Figure 1 for an explanation of the conserved, shaded domains in the c-Myb protein and how they are labeled in the diagrams.

Evidence from two different types of human tumors suggests that loss of the regulatory C-terminal domains of Myb can be a driver mutation leading to oncogenesis. The first mechanism is evident in leukemia samples, in which enhanced alternative RNA splicing produces variant MYB gene transcripts [1,46,47,48,49,50]. Due to the presence of at least six alternative exons plus a number of alternative splice donor and acceptor sites in the standard exons (Figure 3A), the MYB gene can produce more than 60 different mRNA variants that can encode at least 20 different versions of c-Myb protein. All of the alternative RNA splicing occurs in the portion of the gene that encodes the C-terminal regulatory domain; so all of the variant proteins have the normal N-terminal DNA binding domain but different C-terminal structures (Figure 3B). Thus, all of the variants should be able to bind the same DNA sequences, but their targeting to specific genes depends on the unique interactions promoted by their different C-terminal domains. By analyzing MYB alternative RNA splicing in great detail in a small cohort of leukemia samples, it was determined that primary hematopoietic and leukemia cells can produce a diverse set of MYB transcripts that show cell type specificity, that different variants have distinct transcriptional activities and that the expression of some variants correlates with poor patient survival [47]. These results support the hypothesis that enhanced alternative RNA splicing in leukemias leads to the production of truncated, oncogenic variants of c-Myb protein that contribute to leukemogenesis. However, there is not yet direct evidence that variant forms of c-Myb produced as a result of alternative RNA splicing actually act as oncogenic drivers in human leukemia.

The second and best evidence that Myb proteins play a driver role in human oncogenesis comes from the discovery of recurrent t(6;9) translocations in Adenoid Cystic Carcinoma that fuse the MYB gene on chromosome 6 to the NFIB gene on chromosome 9 [51,52]. The gene fusions produce truncated c-Myb proteins lacking the C-terminal domains, reminiscent of the v-Myb proteins encoded by the avian retroviruses AMV and E26 and of the variant Myb proteins produced by leukemias as a result of alternative RNA splicing (Figure 3B). The common theme is that truncation of the C-terminal domain of c-Myb leads to its oncogenic activation. What remains to be explained is what are the unique activities of the truncated Myb proteins, how do they contribute to oncogenesis, and can they be targeted in some way to produce novel therapeutic approaches?

## 2. Myb as a Cell Cycle-Regulated Transcription Factor

### 2.1. Links between Myb and Cell Cycle Regulation

#### 2.1.1. Myb Proteins, Cyclins and CDKs

As a proto-oncoprotein with the potential to induce cell proliferation, c-Myb is likely to play a role in stimulating progression through the cell cycle in normal cells. Myb proteins interact with cell cycle regulators such as Cyclin D1, and the interactions are different for c-Myb compared to oncogenic v-Myb, suggesting that interactions with cell cycle regulators may be important for oncogenic activity [53]. In human cells, c-Myb protein interacts with and is regulated by Cyclin D1 and CDK4 or CDK6, suggesting that regulation of c-Myb activity may play a role in the G1/S transition [54]. In contrast, c-Myb has also been shown to directly regulate the promoter of the Cyclin B1 gene, which is involved in G2/M phase transition [55]. These results could suggest that c-Myb is important for multiple cell cycle regulatory events, in both G1 and G2, and early results studying the cell cycle effects of inactivating temperature-sensitive v-Myb proteins led to similar conclusions [56]. Whole genome studies have shown that cell cycle regulated promoters have conserved motifs, including Myb binding sites [57,58], consistent with a role for Myb proteins in cell cycle regulation.

#### 2.1.2. Myb Regulation of Genes that Regulate the Cell Cycle

An important target gene that can be regulated by c-Myb is the MYC oncogene, which is involved in many types of human cancer [59,60]. Despite their similar names, the Myb and Myc proteins are structurally unrelated, although both are DNA-binding transcription factors with oncogenic activities. However, several early studies identified c-Myb binding sites in the MYC gene promoter, and showed that c-Myb could activate the MYC promoter in reporter gene assays [61,62]. Later studies confirmed the direct regulation of MYC gene expression by c-Myb using chromatin immunoprecipitation (ChIP) assays [63,64]. Those results have even been confirmed using whole-genome ChIP-chip experiments [65]. However, the regulation of the MYC gene is complex and can be affected by many regulatory elements, including some that are distant from the MYC promoter [66,67,68]. So while c-Myb can affect the expression of the MYC gene, Myb is not required for MYC gene expression in all situations, nor is it involved in regulation of the MYC gene in all cell types.

The c-Myb protein has also been implicated in the regulation of genes that control the cell cycle, including the CCNB1 and CCNE1 genes, which encode the Cyclin B1 and Cyclin E1 proteins, respectively [55,69,70]. These results have been confirmed in other cell types using ChIP assays [71]. Interestingly, the related protein B-Myb has been implicated in the regulation of the CCND1 gene, which encodes the G1/S regulator Cyclin D1 [72,73]. So both c-Myb and B-Myb appear to be involved in cell cycle regulation, at least indirectly. A major question that remains is how the Myb proteins are themselves controlled so that these important regulators get expressed at the correct times in the cell cycle? This implies that the activities of the c-Myb and B-Myb proteins are likely to be controlled during the cell cycle, and that they may be active in some parts of the cell cycle and inactive in others.

#### 2.1.3. Retargeting Myb to Different Promoters during the Cell Cycle

The c-Myb protein interacts with the cell cycle regulator Cyclin D1 [53] and its transcriptional activity is subject to control by Cyclin D1/CDK4/6 and by the Cyclin-Dependent Kinase inhibitor p27Kip1 [54]. In addition, c-Myb binds to the promoter of the Cyclin B1 gene and regulates its expression in the G2/M phase of the cell cycle [55]. But what happens to Myb during the other parts of the cell cycle? Does it remain bound to the Cyclin B1 promoter even when the gene is not expressed? Or does the specificity of Myb change dynamically during the cell cycle, moving to different target genes as it interacts with different co-regulators in response to upstream signals?

**Figure 4:**
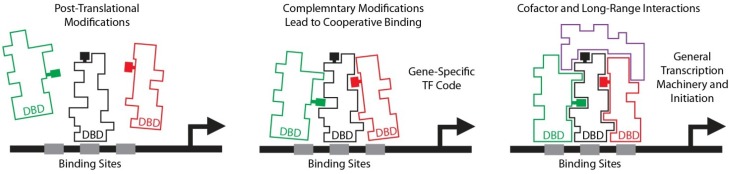
Transcription Factor Interactions. In this model promoters bind multiple transcription factors that must interact to form stable complexes that can stimulate gene expression. Post-translational modifications on the individual transcription factors (left) are the result of upstream signaling pathways, which promote interactions (middle), forming co-operative and synergistic binding and leading to interactions with co-activators and productive transcription initiation (right).

This question was addressed by developing an assay that allowed Myb to be fixed to its cognate promoters, followed by fluorescence activated cell sorting to enrich cells in different phases of the cell cycle. The fixed chromatin was then purified and incubated with Myb-specific antibodies to complete the chromatin-immunoprecipitation (ChIP) assay. The results showed that Myb proteins associate with different promoters in different parts of the cell cycle [71]. For example, Myb associates with the Cyclin B1 promoter in G2/M, and the CXCR4 gene promoter in S and G2 [71]. The Myb DNA binding domains do not change during the cell cycle, nor do the DNA sequences that Myb proteins recognize in promoters. So the differences must occur in the interactions between Myb and other transcription factors or in co-activators that form multi-protein complexes at the promoters of regulated genes (Figure 4).

### 2.2. Regulation of B-Myb in the Cell Cycle

The c-Myb protein is closely related to another transcription factor, B-Myb, the product of the gene MYBL2. Although both B- and c-Myb share homology in their DNA binding domains, they activate different sets of genes [1,4,5,6,74,75,76,77]. Expression of B-Myb is very tightly linked to the cell cycle. B-Myb is able to activate genes responsible for promoting entry into the S- and M-phases of the cell cycle [73,77,78] and can also stimulate expression of genes that are expressed in the G2/M-phase of the cell cycle [78,79]. The transcriptional activity of the B-Myb protein is tightly regulated, and is stimulated through phosphorylation by Cyclin A/CDK2 [78,80,81]. B-Myb is an essential component of the LINC/DREAM complex, an important regulator of cell division, which controls the expression of G2/M-specific genes [78,82,83,84], many of which have been identified in chromatin immunoprecipitation experiments [85]. Overexpression of B-Myb occurs in several aggressive types of cancer [78,86,87,88], suggesting that B-Myb plays a role in tumorigenesis, or at least in the regulation of cell division in the tumor cells. Finally, repression of B-Myb causes cells to undergo premature senescence [78,89,90], and some studies have also revealed that B-Myb is capable of repressing gene expression by competition with other transcription factors [78,83,91]. Thus, developing therapeutic agents that target B-Myb could provide a novel approach for blocking the growth of rapidly dividing tumor cells.

### 2.3. Contrasting Roles of B-Myb and c-Myb in Cell Cycle Regulation

The c-Myb and B-Myb proteins both appear to be regulated through phosphorylation by Cyclin/CDK complexes, but may be specific for different phases of the cell cycle. Both proteins have an N-terminal DNA binding domain and a large C-terminal domain that provides negative regulation and specificity (Figure 1). Both proteins may be regulated through intra-molecular interactions that are controlled by cell cycle-specific phosphorylation [1]. For example, in response to phosphorylation by Cyclin D1/CDK4/6, c-Myb may activate genes required for the G1/S phase transition. In contrast, B-Myb is regulated by Cyclin A/CDK2 in G2/M, and appears to control the late phases of the cell cycle. This difference could explain why c-Myb is a proto-oncogene, capable of stimulating normal cells to enter or progress faster through the cell cycle, while B-Myb, which is required for cell division, is not oncogenic since it cannot initiate the change from resting state to cell cycle progression.

## 3. Situation-Specific Activities of Myb Proteins

### 3.1. Context-Specific Activities of Myb

#### 3.1.1. Myb Activities during Differentiation

The cell cycle regulation of c-Myb described above suggests that its activity is regulated through post-translational modifications. The specificity of the c-Myb transcription factor is also regulated during hematopoietic cell differentiation. The c-Myb protein is relatively highly expressed in immature, dividing hematopoietic cells of both the myeloid and lymphoid lineages, and expression levels decline as the cells differentiate [1,7]. Although c-Myb protein activity is not required for the initial hematopoiesis that occurs in the yolk sac, it is essential for fetal liver hematopoiesis and homozygous knockout of the MYB gene leads to embryonic lethality, characterized by a catastrophic defect in hematopoiesis [92]. Mouse embryos lacking c-Myb protein fail to develop past the stage when fetal liver hematopoiesis begins. More recent, targeted knockout experiments have shown that c-Myb is required for the development of most myeloid and erythroid lineages [93,94,95] and for both B- and T-cell development [96,97,98,99]. The requirement for c-Myb activity in hematopoiesis was also discovered in mutational screens that identified point mutants in c-Myb causing decreased hematopoiesis [100], which will be discussed more in the next section.

Although Myb is sometimes described as being specific for hematopoietic lineages, it is actually involved in the differentiation, development and maintenance of many non-hematopoietic cell types, especially epithelial cells in the gut, kidney and mammary gland [42,65,101,102], as well as smooth muscle cells [103] and some neural stem cells [104]. Myb proteins are thought to be regulated by Wnt signaling pathways, and may play an important role in changes in gene expression that occur during aging [105]. Thus, the c-Myb protein appears to be involved in regulating gene expression and differentiation in a wide variety of tissues and lineages. However, microarray experiments have shown that overexpressed c-Myb activates different genes in different cell types, suggesting that the choice of target genes is determined, at least in part, by the cellular context [11,15]. Myb likely works together with other cell-type specific co-regulators to activate target genes. The presence or absence of different co-regulators in different cell types determines which target genes can be regulated by Myb. This concept led to a model for how Myb proteins could be regulated during differentiation and in different tissues, through a protein-protein interaction code [5]. As shown in Figure 4, the interactions between Myb and other transcription factors are likely regulated by post-translational modifications that stabilize or disrupt interactions, and guide Myb (and other transcription factors) to make stable complexes at specific promoters. Thus, mutations in Myb that alter these protein-protein interactions, such as the mutations in the oncogenic variants like v-Myb, would lead to changes in which target genes are regulated. Similarly, changes in signal transduction pathways that lead to different post-translational modifications in Myb, or the co-regulators, would also lead to changes in gene expression. These types of changes could also occur during the cell cycle, allowing Myb to regulate different target genes in different parts of the cell cycle because of stage-specific changes in post-translational modifications.

#### 3.1.2. Combinatorial Interactions between Myb and Other Transcription Factors

The first-identified Myb-regulated gene, *mim-1*, provides an excellent example of how Myb proteins co-operate with other transcription factors to regulate genes in a cell type-specific manner. The c-Myb and v-Myb proteins bind a high-affinity site in the *mim-1* gene promoter and strongly activate the promoter in reporter gene assays [24]. However, activation of the endogenous, chromatin-embedded *mim-1* gene requires the combination of c-Myb plus another transcription factor, C/EBPbeta (also called NF-M), which binds at an adjacent site [106]. The *mim-1* gene is regulated by both transcription factors and only in the cells where both are active. The c-Myb protein is primarily expressed in hematopoietic and epithelial tissues. The C/EBPbeta protein is restricted to a few tissues such as liver and some myeloid cell lineages. The *mim-1* gene is only expressed in a subset of myeloid cells that express both c-Myb and C/EBPbeta. However, an artificial combination of c-Myb plus C/EBPbeta is sufficient to activate the endogenous *mim-1* gene in other cell types. For example, ectopic expression of c-Myb plus C/EBPbeta is sufficient to activate expression of the gene in cells such as fibroblasts or lymphoid cells where *mim-1* is usually not expressed [106]. The *mim-1* gene appears to be especially responsive to c-Myb and C/EBPbeta because the two transcription factors bind not only the *mim-1* promoter, but also a key upstream enhancer [107]. Ectopic expression of the c-Myb and C/EBPbeta proteins leads to activation of the enhancer, reorganization of the chromatin around the *mim-1* gene and activation of the promoter [107,108,109], allowing the gene to respond to the combination of c-Myb and C/EBPbeta in a wide variety of cell types and demonstrating how a relatively simple transcription factor code (Myb plus C/EBPbeta) can lead to tissue-specific gene regulation.

#### 3.1.3. Myb Interactions with CBP and p300

The c-Myb protein has also been shown to interact and co-operate with the transcriptional co-activator CREB-binding protein, or CBP, and the highly related protein p300. Both CBP and p300 have conserved protein-protein interaction domains called KIX domains that bind the Myb transcriptional activation domain (TAD) [110,111,112,113]. CBP and p300 have histone acetyltransferase activities and also acetylate Myb when they interact with it [110,111]. Acetylation could alter the specificity of Myb by altering its protein-protein interactions, helping to determine which target genes get regulated. Genetic evidence suggests that a specific interaction between Myb and CBP is required for normal hematopoiesis. A mutagenesis screen in mice identified defects in hematopoietic stem cell differentiation caused by a mutation in Myb, M303V, which disrupts the interaction between Myb and p300 [100]. The mutation led to a large increase in the number of immature hematopoietic stem cells in the bone marrow, with concomitant defects in the production of T-cells, B-cells, erythroid cells and myeloid cells, suggesting that an interaction between Myb and p300 is critical for the earliest stages of hematopoietic stem cell differentiation and for normal hematopoiesis [100]. Complementary studies showed that both p300 and CBP play a role in hematopoiesis and that both interact with Myb proteins, although some hematopoietic lineages may depend more on p300 [112]. These studies reaffirmed that Myb activity is required for cell differentiation, which is often thought to be the opposite of transformation.

CBP and p300 interact with a number of transcription factors besides Myb, including GATA-1, FOXO proteins, C/EBPbeta, ETS-1, NFATc4, RelA, E2a-PBX1 and TP53 [114,115,116,117,118,119], mostly through the same conserved domain known as the KIX domain. The interactions between the KIX domain of CBP/p300 and Myb have been studied in great detail [113,120], and could potentially be targeted by drugs designed to disrupt the interactions. Interestingly, the KIX domain is able to interact with Myb and with the Mixed Lineage Leukemia (MLL) protein simultaneously, in a three-way complex that may be important for oncogenesis [121,122]. Understanding how these proteins interact, how the interactions affect the choice of target genes that are regulated by Myb, and how these interactions play a role in the development of tumors or leukemia could provide important information for the development of new drugs or therapeutic strategies.

### 3.2. Protein-Protein Interactions Regulate Myb Activity

**Figure 5:**
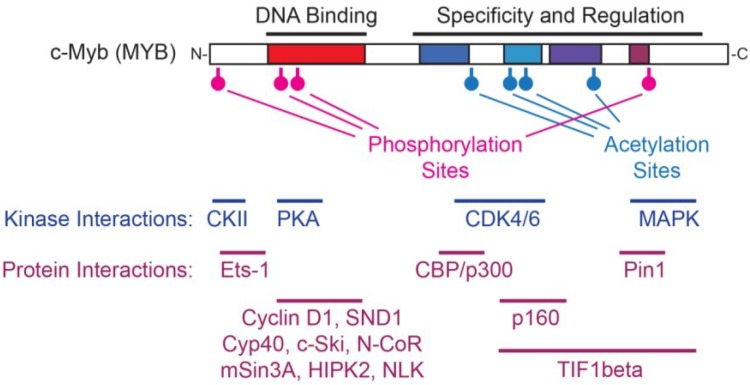
Multiple Regulatory Pathways Lead to Myb. The c-Myb protein is diagrammed at top, with DNA binding domain (red) and C-terminal regulatory domains labeled. Sites of phosphorylation and acetylation are indicated. The lower section shows the approximate binding or interaction sites for kinases or other proteins. Please see the legend to Figure 1 for an explanation of the conserved, shaded domains in the c-Myb protein.

The c-Myb protein has been reported to interact with a large number of potential regulators of its activity, including protein kinases, cell cycle regulators and transcription factors (Figure 5). The c-Myb protein is also subject to a large number of post-translational modifications, including serine and threonine phosphorylation [21,123,124,125,126,127,128,129,130,131], lysine acetylation [110], ubiquitinylation [132] and sumoylation [37,131,133]. Changes in post-translational modifications are likely to alter protein-protein interactions, leading to changes in transcriptional specificity (Figure 4). Since post-translational modifications can occur quickly, often as the result of upstream signaling cascades initiated by cell surface receptors, it seems likely that Myb protein activities and specificities are able to change in response to extracellular signals. This provides a mechanism for Myb proteins to respond to cytokine or growth factor receptors or to cell-cell interactions that control hematopoietic or epithelial cell differentiation. Indeed, chromatin immunoprecipitation experiments have shown that Myb proteins associate with largely different sets of promoters in sparsely growing cells compared to densely plated cells, which make multiple cell-cell contacts [65]. This mechanism may explain how Myb proteins are able to bind different promoters in different cell types, before and after cell-cell interactions or even in different parts of the cell cycle. Unfortunately, detailed proteomics studies that could explain how the Myb protein modifications and interactions change in different situations are still lacking and clearly need to be a priority for future studies.

## 4. Conclusions: Protein Interactions Could Provide Novel Therapeutic Targets

The c-Myb protein appears to be regulated by a large number of post-translational modifications that are likely to affect its interactions with co-regulators like C/EBPbeta and transcriptional co-activators like CBP or p300. In the case of the *mim-1* gene, a simple transcription factor code, Myb plus C/EBPbeta, leads to activation of the gene, even in cells that would otherwise never express *mim-1*. If the example of the *mim-1* gene is typical of Myb-regulated genes, Myb proteins could make a large number of cell type-specific, combinatorial interactions with other transcription factors to regulate different sets of target genes in specific cell types or in specific situations, such as in different parts of the cell cycle. The example of the CCNB1 gene shows that c-Myb can bind to different promoters in different parts of the cell cycle, apparently moving dynamically to different target promoters as it makes stable interactions with different partners. Thus, it is likely that Myb interacts with other transcription factors to regulate specific genes in B-cells and still others to regulate specific genes in colon or breast cells. All of these interactions are subject to regulation by upstream signaling pathways that lead to changes in post-translational modifications and therefore affect protein-protein interactions. These results suggest that each Myb-regulated gene is likely to be controlled by a specific combination of Myb proteins, with appropriate gene-specific post-translational modifications, that interact with unique sets of transcriptional co-regulators that together define a specific transcription factor code for each gene (Figure 4).

Although the simple transcription factor code that regulates the *mim-1* gene may be unusual, it raises the possibility that many genes could be controlled through similar combinations of transcription factors that must interact at promoters or enhancers. These interactions could potentially be targeted by small molecules that disrupt the necessary interactions, or that stabilize alternative ones, in order to alter gene expression patterns. The power of individual protein-protein interactions was demonstrated by the Myb M303V mutation that disrupted interactions with the p300 KIX domain and had a dramatic effect on hematopoietic differentiation. If gene-specific protein-protein interactions could be identified and then targeted with small molecules, new opportunities for regulating individual genes with specific drugs could be created. For example, if Myb proteins interact with p300 to activate proliferation-specific genes, but with co-regulators like C/EBPbeta to regulate differentiation-specific genes, a small molecule that inhibits the former but not the latter could trigger leukemia cells to stop proliferating and to differentiate instead. Although no such small molecule inhibitors of Myb proteins have yet been described, small molecule inhibitors that disrupt the interaction of CBP and p300 with other transcription factors have been identified [134,135]. Identifying similar molecules that disrupt Myb protein functions could lead to important new therapies.

In summary, Myb serves as an excellent model for identifying and studying the complexities of transcriptional regulation in normal and transformed cells. The normal Myb protein regulates different genes than the oncogenic variants. The former is required for normal differentiation while the latter transforms cells and drives tumorigenesis. Both normal and oncogenic forms of Myb have the same DNA binding domain and are capable of binding the same sites in DNA—the difference between them appears to be in the protein-protein interactions they make, which targets them to specific subsets of target genes. Thus, the normal and oncogenic Myb proteins are not only different, but they oppose each other. To adequately treat a tumor driven by an oncogenic Myb it may be necessary to both inactivate the driver and also to reactivate the normal Myb, to induce differentiation. Understanding the transcription factor codes that regulate different Myb target genes could lead to novel therapeutic approaches for turning specific genes on or off, or for inducing tumor cells to differentiate rather than proliferate.

## References

[1] Zhou Y., Ness S.A. (2011). Myb proteins: Angels and demons in normal and transformed cells. Front Biosci. Landmark Ed..

[2] Feller A., Machemer K., Braun E.L., Grotewold E. (2011). Evolutionary and comparative analysis of MYB and bHLH plant transcription factors. Plant J..

[3] Du H., Zhang L., Liu L., Tang X.F., Yang W.J., Wu Y.M., Huang Y.B., Tang Y.X. (2009). Biochemical and molecular characterization of plant MYB transcription factor family. Biochem. Biokhimiia.

[4] Ramsay R.G., Gonda T.J. (2008). MYB function in normal and cancer cells. Nat. Rev. Cancer.

[5] Ness S.A. (2003). Myb protein specificity: Evidence of a context-specific transcription factor code. Blood Cells Mol. Dis..

[6] Lipsick J.S., Manak J., Mitiku N., Chen C.K., Fogarty P., Guthrie E. (2001). Functional evolution of the Myb oncogene family. Blood Cells Mol. Dis..

[7] Ness S.A. (1996). The myb oncoprotein: Regulating a regulator. Biochim. Biophys. Acta.

[8] Lipsick J.S. (1996). One billion years of Myb. Oncogene.

[9] Park D.J., Vuong P.T., de Vos S., Douer D., Koeffler H.P. (2003). Comparative analysis of genes regulated by PML/RAR alpha and PLZF/RAR alpha in response to retinoic acid using oligonucleotide arrays. Blood.

[10] Minucci S., Monestiroli S., Giavara S., Ronzoni S., Marchesi F., Insinga A., Diverio D., Gasparini P., Capillo M., Colombo E. (2002). PML-RAR induces promyelocytic leukemias with high efficiency following retroviral gene transfer into purified murine hematopoietic progenitors. Blood.

[11] Rushton J.J., Davis L.M., Lei W., Mo X., Leutz A., Ness S.A. (2003). Distinct changes in gene expression induced by A-Myb, B-Myb and c-Myb proteins. Oncogene.

[12] Rushton J.J., Ness S.A. (2001). The conserved DNA binding domain mediates similar regulatory interactions for A-Myb, B-Myb, and c-Myb transcription factors. Blood Cells Mol. Dis..

[13] Dini P., Eltman J., Lipsick J. (1995). Mutations in the DNA-binding and transcriptional activation domains of v-Myb cooperate in transformation. J. Virol..

[14] Dini P.W., Lipsick J.S. (1993). Oncogenic truncation of the first repeat of c-Myb decreases DNA binding *in vitro* and *in vivo*. Mol. Cell. Biol..

[15] Lei W., Rushton J.J., Davis L.M., Liu F., Ness S.A. (2004). Positive and negative determinants of target gene specificity in Myb transcription factors. J. Biol. Chem..

[16] Gonda T.J., Cory S., Sobieszczuk P., Holtzman D., Adams J.M. (1987). Generation of altered transcripts by retroviral insertion within the c-Myb gene in two murine monocytic leukemias. J. Virol..

[17] Gonda T.J., Buckmaster C., Ramsay R.G. (1989). Activation of c-Myb by carboxy-terminal truncation: Relationship to transformation of murine haemopoietic cells *in vitro*. EMBO J..

[18] Dubendorff J.W., Whittaker L.J., Eltman J.T., Lipsick J.S. (1992). Carboxy-terminal elements of c-Myb negatively regulate transcriptional activation in cis and in trans. Genes Dev..

[19] Wang D.M., Lipsick J.S. (2002). Mutational analysis of the transcriptional activation domains of v-Myb. Oncogene.

[20] Fu S.L., Lipsick J.S. (1996). FAETL motif required for leukemic transformation by v-Myb. J. Virol..

[21] Dash A.B., Orrico F.C., Ness S.A. (1996). The EVES motif mediates both intermolecular and intramolecular regulation of c-Myb. Genes Dev..

[22] Leverson J.D., Ness S.A. (1998). Point mutations in v-Myb disrupt a cyclophilin-catalyzed negative regulatory mechanism. Mol. Cell.

[23] Pani E., Menigatti M., Schubert S., Hess D., Gerrits B., Klempnauer K.H., Ferrari S. (2008). Pin1 interacts with c-Myb in a phosphorylation-dependent manner and regulates its transactivation activity. Biochim. Biophys. Acta.

[24] Ness S.A., Marknell A., Graf T. (1989). The v-Myb oncogene product binds to and activates the promyelocyte-specific mim-1 gene. Cell.

[25] Introna M., Golay J., Frampton J., Nakano T., Ness S., Graf T. (1990). Mutations in v-Myb alter the differentiation of myelomonocytic cells transformed by the oncogene. Cell.

[26] Ogata K., Kanai H., Inoue T., Sekikawa A., Sasaki M., Nagadoi A., Sarai A., Ishii S., Nishimura Y. (1993). Solution structures of Myb DNA-binding domain and its complex with DNA. Nucleic Acids Symp. Ser..

[27] Ogata K., Hojo H., Aimoto S., Nakai T., Nakamura H., Sarai A., Ishii S., Nishimura Y. (1992). Solution structure of a DNA-binding unit of Myb: A helix-turn-helix-related motif with conserved tryptophans forming a hydrophobic core. Proc. Natl. Acad. Sci. USA.

[28] Tahirov T.H., Morii H., Uedaira H., Sarai A., Ogata K. (1999). Crystallization and preliminary X-ray analysis of wild-type and V103L mutant Myb R2 DNA-binding domain. Acta Crystallogr. D Biol. Crystallogr..

[29] Boyer L.A., Langer M.R., Crowley K.A., Tan S., Denu J.M., Peterson C.L. (2002). Essential role for the SANT domain in the functioning of multiple chromatin remodeling enzymes. Mol. Cell.

[30] Sterner D.E., Wang X., Bloom M.H., Simon G.M., Berger S.L. (2002). The SANT domain of Ada2 is required for normal acetylation of histones by the yeast SAGA complex. J. Biol. Chem..

[31] Boyer L.A., Latek R.R., Peterson C.L. (2004). The SANT domain: A unique histone-tail-binding module?. Nat. Rev. Mol. Cell Biol..

[32] Humphrey G.W., Wang Y., Russanova V.R., Hirai T., Qin J., Nakatani Y., Howard B.H. (2001). Stable histone deacetylase complexes distinguished by the presence of SANT domain proteins CoREST/kiaa0071 and Mta-L1. J. Biol. Chem..

[33] Zaret K.S., Watts J., Xu J., Wandzioch E., Smale S.T., Sekiya T. (2008). Pioneer factors, genetic competence, and inductive signaling: Programming liver and pancreas progenitors from the endoderm. Cold Spring Harb. Symp. Quant. Biol..

[34] Zaret K.S., Carroll J.S. (2011). Pioneer transcription factors: Establishing competence for gene expression. Genes Dev..

[35] Mo X., Kowenz-Leutz E., Laumonnier Y., Xu H., Leutz A. (2005). Histone H3 tail positioning and acetylation by the c-Myb but not the v-Myb DNA-binding SANT domain. Genes Dev..

[36] Nomura T., Tanikawa J., Akimaru H., Kanei-Ishii C., Ichikawa-Iwata E., Khan M.M., Ito H., Ishii S. (2004). Oncogenic activation of c-Myb correlates with a loss of negative regulation by TIF1beta and Ski. J. Biol. Chem..

[37] Bies J., Markus J., Wolff L. (2002). Covalent attachment of the SUMO-1 protein to the negative regulatory domain of the c-Myb transcription factor modifies Its stability and transactivation capacity. J. Biol. Chem..

[38] Hu Y.L., Ramsay R.G., Kanei-Ishii C., Ishii S., Gonda T.J. (1991). Transformation by carboxyl-deleted Myb reflects increased transactivating capacity and disruption of a negative regulatory domain. Oncogene.

[39] Liu F., Lei W., OʼRourke J.P., Ness S.A. (2006). Oncogenic mutations cause dramatic, qualitative changes in the transcriptional activity of c-Myb. Oncogene.

[40] Clappier E., Cuccuini W., Kalota A., Crinquette A., Cayuela J.M., Dik W.A., Langerak A.W., Montpellier B., Nadel B., Walrafen P. (2007). The C-MYB locus is involved in chromosomal translocation and genomic duplications in human T-cell acute leukemia (T-ALL), the translocation defining a new T-ALL subtype in very young children. Blood.

[41] Lahortiga I., de Keersmaecker K., van Vlierberghe P., Graux C., Cauwelier B., Lambert F., Mentens N., Beverloo H.B., Pieters R., Speleman F. (2007). Duplication of the MYB oncogene in T cell acute lymphoblastic leukemia. Nat. Genet..

[42] Drabsch Y., Hugo H., Zhang R., Dowhan D., Miao Y., Gewirtz A., Barry S., Ramsay R., Gonda T. (2007). Mechanism of and requirement for estrogen-regulated MYB expression in estrogen-receptor-positive breast cancer cells. Proc. Natl. Acad. Sci. USA.

[43] Thorner A.R., Parker J.S., Hoadley K.A., Perou C.M. (2010). Potential tumor suppressor role for the c-Myb oncogene in luminal breast cancer. PLoS One.

[44] Beug H., Blundell P., Graf T. (1987). Reversibility of differentiation and proliferative capacity in avian myelomonocytic cells transformed by ts E26 leukemia virus. Genes Dev..

[45] Ness S.A., Beug H., Graf T. (1987). v-Myb dominance over v-Myc in doubly transformed chick myelomonocytic cells. Cell.

[46] Zhou Y.E., O’Rourke J.P., Edwards J.S., Ness S.A. (2011). Single molecule analysis of c-Myb alternative splicing reveals novel classifiers for precursor B-ALL. PLoS One.

[47] O’Rourke J.P., Ness S.A. (2008). Alternative RNA splicing produces multiple forms of c-Myb with unique transcriptional activities. Mol. Cell. Biol..

[48] Schuur E.R., Rabinovich J.M., Baluda M.A. (1994). Distribution of alternatively spliced chicken c-Myb exon 9A among hematopoietic tissues. Oncogene.

[49] Shen-Ong G.L., Skurla R.M., Owens J.D., Mushinski J.F. (1990). Alternative splicing of RNAs transcribed from the human c-Myb gene. Mol. Cell. Biol..

[50] Westin E.H., Gorse K.M., Clarke M.F. (1990). Alternative splicing of the human c-Myb gene. Oncogene.

[51] Brill L.B., Kanner W.A., Fehr A., Andren Y., Moskaluk C.A., Loning T., Stenman G., Frierson H.F. (2011). Analysis of MYB expression and MYB-NFIB gene fusions in adenoid cystic carcinoma and other salivary neoplasms. Mod. Pathol..

[52] Persson M., Andren Y., Mark J., Horlings H.M., Persson F., Stenman G. (2009). Recurrent fusion of MYB and NFIB transcription factor genes in carcinomas of the breast and head and neck. Proc. Natl. Acad. Sci. USA.

[53] Ganter B., Fu S., Lipsick J.S. (1998). D-type cyclins repress transcriptional activation by the v-Myb but not the c-Myb DNA-binding domain. EMBO J..

[54] Lei W., Liu F., Ness S.A. (2005). Positive and negative regulation of c-Myb by Cyclin D1, Cyclin-Dependent kinases and p27 Kip1. Blood.

[55] Nakata Y., Shetzline S., Sakashita C., Kalota A., Rallapalli R., Rudnick S., Zhang Y., Emerson S., Gewirtz A. (2007). c-Myb contributes to G2/M cell cycle transition in human hematopoietic cells by direct regulation of cyclin B1 expression. Mol. Cell. Biol..

[56] Frampton J., Ramqvist T., Graf F. (1996). v-Myb of E26 leukemia virus up-regulates bcl-2 and suppresses apoptosis in myeloid cells. Genes Dev..

[57] Wasner M., Haugwitz U., Reinhard W., Tschop K., Spiesbach K., Lorenz J., Mossner J., Engeland K. (2003). Three CCAAT-boxes and a single cell cycle genes homology region (CHR) are the major regulating sites for transcription from the human cyclin B2 promoter. Gene.

[58] Wasner M., Tschop K., Spiesbach K., Haugwitz U., Johne C., Mossner J., Mantovani R., Engeland K. (2003). Cyclin B1 transcription is enhanced by the p300 coactivator and regulated during the cell cycle by a CHR-dependent repression mechanism. FEBS Lett..

[59] Spender L.C., Inman G.J. (2014). Developments in Burkitt’s lymphoma: Novel cooperations in oncogenic MYC signaling. Cancer Manag. Res..

[60] Bretones G., Delgado M.D., Leon J. (2014). Myc and cell cycle control. Biochim. Biophys. Acta.

[61] Nakagoshi H., Kanei-Ishii C., Sawazaki T., Mizuguchi G., Ishii S. (1992). Transcriptional activation of the c-myc gene by the c-Myb and B-Myb gene products. Oncogene.

[62] Cogswell J.P., Cogswell P.C., Kuehl W.M., Cuddihy A.M., Bender T.M., Engelke U., Marcu K.B., Ting J.P. (1993). Mechanism of c-Myc regulation by c-Myb in different cell lineages. Mol. Cell. Biol..

[63] Berge T., Matre V., Brendeford E.M., Saether T., Luscher B., Gabrielsen O.S. (2007). Revisiting a selection of target genes for the hematopoietic transcription factor c-Myb using chromatin immunoprecipitation and c-Myb knockdown. Blood Cells Mol. Dis..

[64] Ciznadija D., Tothill R., Waterman M.L., Zhao L., Huynh D., Yu R.M., Ernst M., Ishii S., Mantamadiotis T., Gonda T.J. (2009). Intestinal adenoma formation and MYC activation are regulated by cooperation between MYB and Wnt signaling. Cell Death Differ..

[65] Quintana A.M., Liu F., O’Rourke J.P., Ness S.A. (2011). Identification and regulation of c-Myb target genes in MCF-7 cells. BMC Cancer.

[66] Shi J., Whyte W.A., Zepeda-Mendoza C.J., Milazzo J.P., Shen C., Roe J.S., Minder J.L., Mercan F., Wang E., Eckersley-Maslin M.A. (2013). Role of SWI/SNF in acute leukemia maintenance and enhancer-mediated Myc regulation. Genes Dev..

[67] Loven J., Hoke H.A., Lin C.Y., Lau A., Orlando D.A., Vakoc C.R., Bradner J.E., Lee T.I., Young R.A. (2013). Selective inhibition of tumor oncogenes by disruption of super-enhancers. Cell.

[68] Wright J.B., Brown S.J., Cole M.D. (2010). Upregulation of c-MYC in cis through a large chromatin loop linked to a cancer risk-associated single-nucleotide polymorphism in colorectal cancer cells. Mol. Cell. Biol..

[69] Cheasley D., Pereira L., Lightowler S., Vincan E., Malaterre J., Ramsay R.G. (2011). Myb controls intestinal stem cell genes and self-renewal. Stem Cells.

[70] Malaterre J., Carpinelli M., Ernst M., Alexander W., Cooke M., Sutton S., Dworkin S., Heath J.K., Frampton J., McArthur G. (2007). c-Myb is required for progenitor cell homeostasis in colonic crypts. Proc. Natl. Acad. Sci. USA.

[71] Quintana A.M., Zhou Y.E., Pena J.J., O’Rourke J.P., Ness S.A. (2011). Dramatic repositioning of c-Myb to different promoters during the cell cycle observed by combining cell sorting with chromatin immunoprecipitation. PLoS One.

[72] Bartusel T., Schubert S., Klempnauer K.H. (2005). Regulation of the cyclin D1 and cyclin A1 promoters by B-Myb is mediated by Sp1 binding sites. Gene.

[73] Joaquin M., Watson R.J. (2003). Cell cycle regulation by the B-Myb transcription factor. Cell. Mol. Life Sci..

[74] Gonda T.J., Leo P., Ramsay R.G. (2008). Estrogen and MYB in breast cancer: Potential for new therapies. Expert Opin. Biol. Ther..

[75] Greig K.T., Carotta S., Nutt S.L. (2008). Critical roles for c-Myb in hematopoietic progenitor cells. Semin. Immunol..

[76] Ramsay R.G., Barton A.L., Gonda T.J. (2003). Targeting c-Myb expression in human disease. Expert Opin. Ther. Targets.

[77] Sala A., Watson R. (1999). B-Myb protein in cellular proliferation, transcription control, and cancer: Latest developments. J. Cell. Physiol..

[78] Martinez I., Dimaio D. (2011). B-Myb, cancer, senescence, and microRNAs. Cancer Res..

[79] Zhu W., Giangrande P.H., Nevins J.R. (2004). E2Fs link the control of G1/S and G2/M transcription. EMBO J..

[80] Johnson L.R., Johnson T.K., Desler M., Luster T.A., Nowling T., Lewis R.E., Rizzino A. (2002). Effects of B-Myb on gene transcription: Phosphorylation-dependent activity and acetylation by p300. J. Biol. Chem..

[81] Robinson C., Light Y., Groves R., Mann D., Marias R., Watson R. (1996). Cell-cycle regulation of B-Myb protein expression: Specific phosphorylation during the S phase of the cell cycle. Oncogene.

[82] Knight A.S., Notaridou M., Watson R.J. (2009). A Lin-9 complex is recruited by B-Myb to activate transcription of G2/M genes in undifferentiated embryonal carcinoma cells. Oncogene.

[83] Mannefeld M., Klassen E., Gaubatz S. (2009). B-Myb is required for recovery from the DNA damage-induced G2 checkpoint in p53 mutant cells. Cancer Res..

[84] Schmit F., Cremer S., Gaubatz S. (2009). LIN54 is an essential core subunit of the DREAM/LINC complex that binds to the cdc2 promoter in a sequence-specific manner. FEBS J..

[85] Sadasivam S., Duan S., DeCaprio J.A. (2012). The MuvB complex sequentially recruits B-Myb and FoxM1 to promote mitotic gene expression. Genes Dev..

[86] Bar-Shira A., Pinthus J.H., Rozovsky U., Goldstein M., Sellers W.R., Yaron Y., Eshhar Z., Orr-Urtreger A. (2002). Multiple genes in human 20q13 chromosomal region are involved in an advanced prostate cancer xenograft. Cancer Res..

[87] Raschella G., Cesi V., Amendola R., Negroni A., Tanno B., Altavista P., Tonini G.P., de Bernardi B., Calabretta B. (1999). Expression of B-Myb in neuroblastoma tumors is a poor prognostic factor independent from MYCN amplification. Cancer Res..

[88] Thorner A.R., Hoadley K.A., Parker J.S., Winkel S., Millikan R.C., Perou C.M. (2009). *In vitro* and *in vivo* analysis of B-Myb in basal-like breast cancer. Oncogene.

[89] Sala A., Calabretta B. (1992). Regulation of BALB/c 3T3 fibroblast proliferation by B-Myb is accompanied by selective activation of cdc2 and cyclin D1 expression. Proc. Natl. Acad. Sci. USA.

[90] Sala A., de Luca A., Giordano A., Peschle C. (1996). The retinoblastoma family member p107 binds to B-Myb and suppresses its autoregulatory activity. J. Biol. Chem..

[91] Marhamati D.J., Bellas R.E., Arsura M., Kypreos K.E., Sonenshein G.E. (1997). A-Myb is expressed in bovine vascular smooth muscle cells during the late G1-to-S phase transition and cooperates with c-myc to mediate progression to S phase. Mol. Cell. Biol..

[92] Mucenski M.L., McLain K., Kier A.B., Swerdlow S.H., Schreiner C.M., Miller T.A., Pietryga D.W., Scott W.J., Potter S.S. (1991). A functional c-Myb gene is required for normal murine fetal hepatic hematopoiesis. Cell.

[93] Emambokus N., Vegiopoulos A., Harman B., Jenkinson E., Anderson G., Frampton J. (2003). Progression through key stages of haemopoiesis is dependent on distinct threshold levels of c-Myb. EMBO J..

[94] Hess J.L., Bittner C.B., Zeisig D.T., Bach C., Fuchs U., Borkhardt A., Frampton J., Slany R.K. (2006). c-Myb is an essential downstream target for homeobox-mediated transformation of hematopoietic cells. Blood.

[95] Vegiopoulos A., Garcia P., Emambokus N., Frampton J. (2006). Coordination of erythropoiesis by the transcription factor c-Myb. Blood.

[96] Allen R.D. (1999). c-Myb is essential for early T cell development. Genes Dev..

[97] Bender T.P., Kremer C.S., Kraus M., Buch T., Rajewsky K. (2004). Critical functions for c-Myb at three checkpoints during thymocyte development. Nat. Immunol..

[98] Fahl S.P., Crittenden R.B., Allman D., Bender T.P. (2009). c-Myb is required for pro-B cell differentiation. J. Immunol..

[99] Thomas M.D., Kremer C.S., Ravichandran K.S., Rajewsky K., Bender T.P. (2005). c-Myb is critical for B cell development and maintenance of follicular B cells. Immunity.

[100] Sandberg M.L., Sutton S.E., Pletcher M.T., Wiltshire T., Tarantino L.M., Hogenesch J.B., Cooke M.P. (2005). c-Myb and p300 regulate hematopoietic stem cell proliferation and differentiation. Dev. Cell.

[101] Lu B.C., Cebrian C., Chi X., Kuure S., Kuo R., Bates C.M., Arber S., Hassell J., MacNeil L., Hoshi M. (2009). Etv4 and Etv5 are required downstream of GDNF and Ret for kidney branching morphogenesis. Nat. Genet..

[102] Zorbas M., Sicurella C., Bertoncello I., Venter D., Ellis S., Mucenski M.L., Ramsay R.G. (1999). c-Myb is critical for murine colon development. Oncogene.

[103] Kolodziejska K.M., Noyan-Ashraf M.H., Nagy A., Bacon A., Frampton J., Xin H.B., Kotlikoff M.I., Husain M. (2008). c-Myb-dependent smooth muscle cell differentiation. Circ. Res..

[104] Malaterre J., Mantamadiotis T., Dworkin S., Lightowler S., Yang Q., Ransome M.I., Turnley A.M., Nichols N.R., Emambokus N.R., Frampton J. (2008). c-Myb is required for neural progenitor cell proliferation and maintenance of the neural stem cell niche in adult brain. Stem Cells.

[105] Hofmann J.W., McBryan T., Adams P.D., Sedivy J.M. (2014). The effects of aging on the expression of Wnt pathway genes in mouse tissues. Age.

[106] Ness S.A., Kowenz-Leutz E., Casini T., Graf T., Leutz A. (1993). Myb and NF-M: Combinatorial activators of myeloid genes in heterologous cell types. Genes Dev..

[107] Chayka O., Kintscher J., Braas D., Klempnauer K.H. (2005). v-Myb mediates cooperation of a cell-specific enhancer with the mim-1 promoter. Mol. Cell. Biol..

[108] Yamkamon V., Ivanova O., Braas D., Chayka O., Patmasiriwat P., Klempnauer K.H. (2008). A dual activation mechanism for Myb-responsive genes in myelomonocytic cells. Blood Cells Mol. Dis..

[109] Wilczek C., Chayka O., Plachetka A., Klempnauer K.H. (2009). Myb-induced chromatin remodeling at a dual enhancer/promoter element involves non-coding rna transcription and is disrupted by oncogenic mutations of v-Myb. J. Biol. Chem..

[110] Tomita A., Towatari M., Tsuzuki S., Hayakawa F., Kosugi H., Tamai K., Miyazaki T., Kinoshita T., Saito H. (2000). c-Myb acetylation at the carboxyl-terminal conserved domain by transcriptional co-activator p300. Oncogene.

[111] Dai P., Akimaru H., Tanaka Y., Hou D.X., Yasukawa T., Kanei-Ishii C., Takahashi T., Ishii S. (1996). CBP as a transcriptional coactivator of c-Myb. Genes Dev..

[112] Kasper L.H., Fukuyama T., Lerach S., Chang Y., Xu W., Wu S., Boyd K.L., Brindle P.K. (2013). Genetic interaction between mutations in c-Myb and the KIX domains of CBP and p300 affects multiple blood cell lineages and influences both gene activation and repression. PLoS One.

[113] Zor T., de Guzman R.N., Dyson H.J., Wright P.E. (2004). Solution structure of the KIX domain of CBP bound to the transactivation domain of c-Myb. J. Mol. Biol..

[114] Bayly R., Chuen L., Currie R.A., Hyndman B.D., Casselman R., Blobel G.A., LeBrun D.P. (2004). E2A-PBX1 interacts directly with the KIX domain of CBP/p300 in the induction of proliferation in primary hematopoietic cells. J. Biol. Chem..

[115] Denis C.M., Chitayat S., Plevin M.J., Wang F., Thompson P., Liu S., Spencer H.L., Ikura M., LeBrun D.P., Smith S.P. (2012). Structural basis of CBP/p300 recruitment in leukemia induction by E2A-PBX1. Blood.

[116] Mink S., Haenig B., Klempnauer K.H. (1997). Interaction and functional collaboration of p300 and C/EBPbeta. Mol. Cell. Biol..

[117] Mukherjee S.P., Behar M., Birnbaum H.A., Hoffmann A., Wright P.E., Ghosh G. (2013). Analysis of the RelA:CBP/p300 interaction reveals its involvement in NF-kappaB-driven transcription. PLoS Biol..

[118] Wang F., Marshall C.B., Li G.Y., Yamamoto K., Mak T.W., Ikura M. (2009). Synergistic interplay between promoter recognition and CBP/p300 coactivator recruitment by FOXO3a. ACS Chem. Biol..

[119] Yang C., Shapiro L.H., Rivera M., Kumar A., Brindle P.K. (1998). A role for CREB binding protein and p300 transcriptional coactivators in Ets-1 transactivation functions. Mol. Cell. Biol..

[120] Toto A., Giri R., Brunori M., Gianni S. (2014). The mechanism of binding of the KIX domain to the Mixed Lineage Leukemia protein and its allosteric role in the recognition of c-Myb. Protein Sci..

[121] Goto N.K., Zor T., Martinez-Yamout M., Dyson H.J., Wright P.E.  (2002). Cooperativity in transcription factor binding to the coactivator CREB-binding protein (CBP). The mixed lineage leukemia protein (MLL) activation domain binds to an allosteric site on the KIX domain. J. Biol. Chem..

[122] Arai M., Dyson H.J., Wright P.E. (2010). Leu628 of the KIX domain of CBP is a key residue for the interaction with the MLL transactivation domain. FEBS Lett..

[123] Luscher B., Christenson E., Litchfield D.W., Krebs E.G., Eisenman R.N. (1990). Myb DNA binding inhibited by phosphorylation at a site deleted during oncogenic activation. Nature.

[124] Lüscher B., Eisenman R.N. (1992). Mitosis-specific phosphorylation of the nuclear oncoproteins Myc and Myb. J. Cell Biol..

[125] Aziz N., Wu J., Dubendorff J.W., Lipsick J.S., Sturgill T.W., Bender T.P. (1993). c-Myb and v-Myb are differentially phosphorylated by p42mapk *in vitro*. Oncogene.

[126] Bousset K., Oelgeschlager M.H., Henriksson M., Schreek S., Burkhardt H., Litchfield D.W., Luscher-Firzlaff J.M., Luscher B. (1994). Regulation of transcription factors c-Myc, Max, and c-Myb by casein kinase II. Cell. Mol. Biol. Res..

[127] Aziz N., Miglarese M.R., Hendrickson R.C., Shabanowitz J., Sturgill T.W., Hunt D.F., Bender T.P. (1995). Modulation of c-Myb-induced transcription activation by a phosphorylation site near the negative regulatory domain. Proc. Natl. Acad. Sci. USA.

[128] Oelgeschlager M., Krieg J., Luscher-Firzlaff J.M., Luscher B. (1995). Casein kinase II phosphorylation site mutations in c-Myb affect DNA binding and transcriptional cooperativity with NF-M. Mol. Cell. Biol..

[129] Ramsay R.G., Morrice N., van Eeden P., Kanagasundaram V., Nomura T., de Blaquiere J., Ishii S., Wettenhall R. (1995). Regulation of c-Myb through protein phosphorylation and leucine zipper interactions. Oncogene.

[130] Winn L.M., Lei W., Ness S.A. (2003). Pim-1 phosphorylates the DNA binding domain of c-Myb. Cell Cycle.

[131] Bies J., Sramko M., Wolff L. (2013). Stress-induced Phosphorylation of Thr486 in c-Myb by p38MAPKs Attenuates Conjugation of SUMO-2/3. J. Biol. Chem..

[132] Kitagawa K., Kotake Y., Hiramatsu Y., Liu N., Suzuki S., Nakamura S., Kikuchi A., Kitagawa M. (2010). GSK3 regulates the expressions of human and mouse c-Myb via different mechanisms. Cell Div..

[133] Sramko M., Markus J., Kabát J., Wolff L., Bies J. (2006). Stress-induced inactivation of the c-Myb transcription factor through conjugation of SUMO-2/3 proteins. J. Biol. Chem..

[134] Best J.L., Amezcua C.A., Mayr B., Flechner L., Murawsky C.M., Emerson B., Zor T., Gardner K.H., Montminy M. (2004). Identification of small-molecule antagonists that inhibit an activator: Coactivator interaction. Proc. Natl. Acad. Sci. USA.

[135] Sun H., Chung W.C., Ryu S.H., Ju Z., Tran H.T., Kim E., Kurie J.M., Koo J.S. (2008). Cyclic AMP-responsive element binding protein- and nuclear factor-kappaB-regulated CXC chemokine gene expression in lung carcinogenesis. Cancer Prev. Res. (Phila).

